# Disposable Printed Electrode Made with Chinese Shellac and Carbon Black for Melatonin Detection

**DOI:** 10.1021/acsmeasuresciau.5c00056

**Published:** 2025-07-18

**Authors:** Ana Luiza Molina de Cezar, Rafaela Cristina Freitas, Amanda Neumann, Bruno Campos Janegitz

**Affiliations:** Laboratory of Sensors, Nanomedicine, and Nanostructured Materials, 67828 Universidade Federal de São Carlos, Araras, São Paulo 13604-900, Brasil

**Keywords:** disposable screen-printed electrode, melatonin, conductive ink, Chinese shellac, carbon black

## Abstract

Screen-printed electrodes (SPEs) are an innovative technology in electrochemical sensors, offering advantages such as easy fabrication, large-scale production, low cost, and potential for miniaturization. These electrodes can be disposable and customized for various applications. Due to these advantages, SPEs are gaining attention in fields such as medicine and pharmacy. In this study, an electrochemical sensor was developed through screen-printing, using new conductive ink, compounded with carbon black, Chinese shellac, and acetone. The device was characterized by different approaches to analyze its characteristics, including scanning electron microscopy, Fourier transform infrared spectroscopy, X-ray diffraction, thermogravimetry, and contact angle. Also, the electrochemical characterizations were performed by using cyclic voltammetry and impedance spectroscopy. The sensor was employed to detect melatonin, a sleep-regulating hormone, and, under optimized parameters, the analytical curve by differential pulse voltammetry exhibited a linear range from 1.0 to 100 μmol L^–1^, with a limit of detection of 0.1 μmol L^–1^. The device was applied to synthetic urine samples using the addition and recovery method, yielding recovery values from 86.7 to 110%. The results indicate that the conductive ink is suitable for manufacturing printed electrodes, and the device proved promising for melatonin detection.

## Graphical abstract

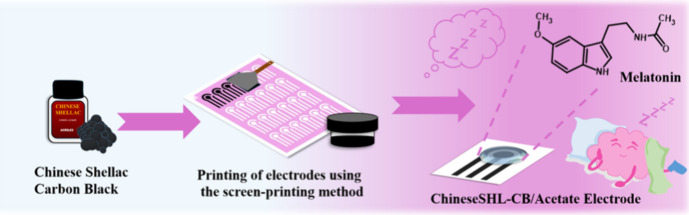

## Introduction

Screen-printed electrodes (SPEs) have emerged as a growing technology in the field of electrochemical sensors, offering advances such as relatively low-cost, ease of production, large-scale manufacturing, and replicability.^1–3^ Thereby, SPEs have become increasingly prevalent in various fields, including medicine, pharmacy, agriculture, and environmental monitoring.^4^ Furthermore, the selection of different materials, like nanomaterials, allows the customization of sensors, tailoring them to meet specific needs.^5,6^ In this context, SPEs represent a valuable tool for the development of new devices and innovative solutions for the detection of chemical substances.^7,8^

Otherwise, conductive inks are composites used to print electrical circuits on surfaces, composed of conductive particles dispersed in a binder, and applied through techniques such as screen-printing.^9,10^ These inks provide the production of electrodes quickly, allowing the printing of electronic devices directly in different substrates, like flexible plastic materials, such as polycarbonate, polyamide, or acetate.^11,12^ Among conductive particles, carbon-based materials have been highlighted, such as carbon black (CB), for its high specific surface area, conductive properties, and relatively low-cost.^6^ These particles stand out in flexible electronics for enabling large-scale production at low-cost, which is desirable for SPEs manufacture.^7,13–15^

Chinese shellac is a natural material extracted from the sap of the shellac tree (*Toxicodendron vernicifluum*), which is widely found in East Asia. Its main component, urushiol, is a molecule composed of long aliphatic chains and aromatic hydrocarbons.^16,17^ This substance undergoes oxidation and polymerization to form a highly durable coating. This transformation results in a three-dimensional network with properties that make it ideal for a wide range of applications. In addition to its chemical and thermal resistance, shellac offers excellent flexibility and adhesion to various substrates.^18^ It is also a sustainable alternative, being renewable and possibly biodegradable.^16,18,19^ These characteristics set Chinese shellac as a promising option for advanced and sustainable devices.^19–21^

As proof of concept, melatonin was chosen for determination for being a hormone secreted by the pineal gland, which presents physiological functions, such as sleep regulation,^22–24^ aiding in the treatment of insomnia, and assisting with circadian rhythm disorders and mental health.^25–27^ Various techniques are used for melatonin detection, including electrochemical methods, and are considered effective for rapid, portable detection at relatively low costs compared to conventional techniques.^28^ Literature includes studies utilizing electrochemical sensors, such as the work by Lete and collaborators^29^ who developed an electrochemical sensor modified with gold nanoparticles on printed carbon electrodes. Similarly, the work of Gomez and collaborators^30^ developed a sensor based on carbon nanotubes or graphene showing high sensitivity for analysis in biological samples.

This study introduces the development of a novel and environmentally friendly conductive ink, formulated using Chinese shellac and carbon black (CB), specifically engineered for the fabrication of cost-effective, disposable screen-printed electrodes (SPEs). The resulting electrochemical sensor was successfully employed for the sensitive and selective quantification of melatonin in synthetic urine samples, utilizing square wave voltammetry (SWV) as the detection technique.

## Experimental Section

### Reagents and Solutions

The reagents were of analytical grade or high purity, purchased from Sigma-Aldrich or Fluka. All aqueous solutions were prepared using ultrapure water processed through a Millipore Milli-Q system (resistivity ≥ 18.2 MΩ cm^–1^). The CB powder was obtained from VULCAN XC72 carbon black by CABOT (Boston, Massachusetts, USA), Chinese shellac/maleic resin (Acrilex, SBC, SP), and melatonin (Alfa Aesar, Massachusetts, USA). Electrochemical analyses were performed using a 3-electrode system (working, pseudoreference, and auxiliary). Electrochemical characterization was carried out using the electrochemical probe ferrocenemethanol (FcMeOH), in 0.1 mol L^–1^ KCl solution. A phosphate buffer saline 0.2 mol L^–1^ PBS at pH 7.0 was used for melatonin determination and sensor optimization, with the salt’s sodium hydrogen phosphate (NaHPO_4_·7H_2_O) and monobasic potassium phosphate (KH_2_PO_4_) being employed.

### Apparatus

The ink was homogenized in a double asymmetric centrifuge, SpeedMixer Dac 150.1 FVZ-K (FlackTec Inc.). All electrochemical measurements were performed with an Autolab PGSTAT204 potentiostat/galvanostat (Eco Chemie) (with FRA32 M module), controlled by NOVA 2.1.3 software. A pH meter 827 (Metrohm) was used for pH measurements. The characterizations of the composite and materials was performed using Scanning Electron Microscopy with field emission, employing a scanning electron microscope (Thermo Fisher Scientific, Prisma E) equipped with an Everhart-Thornley SE detector, Fourier Transform Infrared Spectrophotometer (Bruker, TENSOR II) — MULTIUSER, X-ray Powder Diffractometer (Rigaku, MiniFlex 600) — MULTIUSER, and PerkinElmer TGA 4000 thermogravimetric analyzer (Norwalk, Connecticut, United States). The contact angle analysis were carried out adding 100 μL of deionized water and using a lab-made equipment described in reference.^31^

### Preparation of the Sensors

The conductive ink was prepared using 83.33% of Chinese shellac and 16.67% of carbon black (CB), with 100 μL of acetone as an additive (Figure 1-I). This specific formulation, corresponding to 20% (w/w) CB relative to the shellac mass, was selected after evaluating other concentrations (5% (w/w), 10% (w/w), and 15%n (w/w). The 20% CB ink showed the best electrochemical performance. The resulting mixture, Figure 1-II, was homogenized for 3 cycles of 3500 rpm for 90 s in a double asymmetric SpeedMixer, Figure 1-III. The ink was then applied on the sanded transparent acetate, using an adhesive paper sheet with the design outlined by a Silhouette Cameo 4 cutting printer, Figure 1-IV. After the ink dried for 30 min at room temperature, the SPE was prepared to use for the electrochemical analyses, Figure V. This device was denominated as ChineseSHL-CB/Acetate, and its image is shown in the Supporting Information, in Figure S1, in real proportions of approximately 2.0 per 1.0 cm.

**Figure 1:**
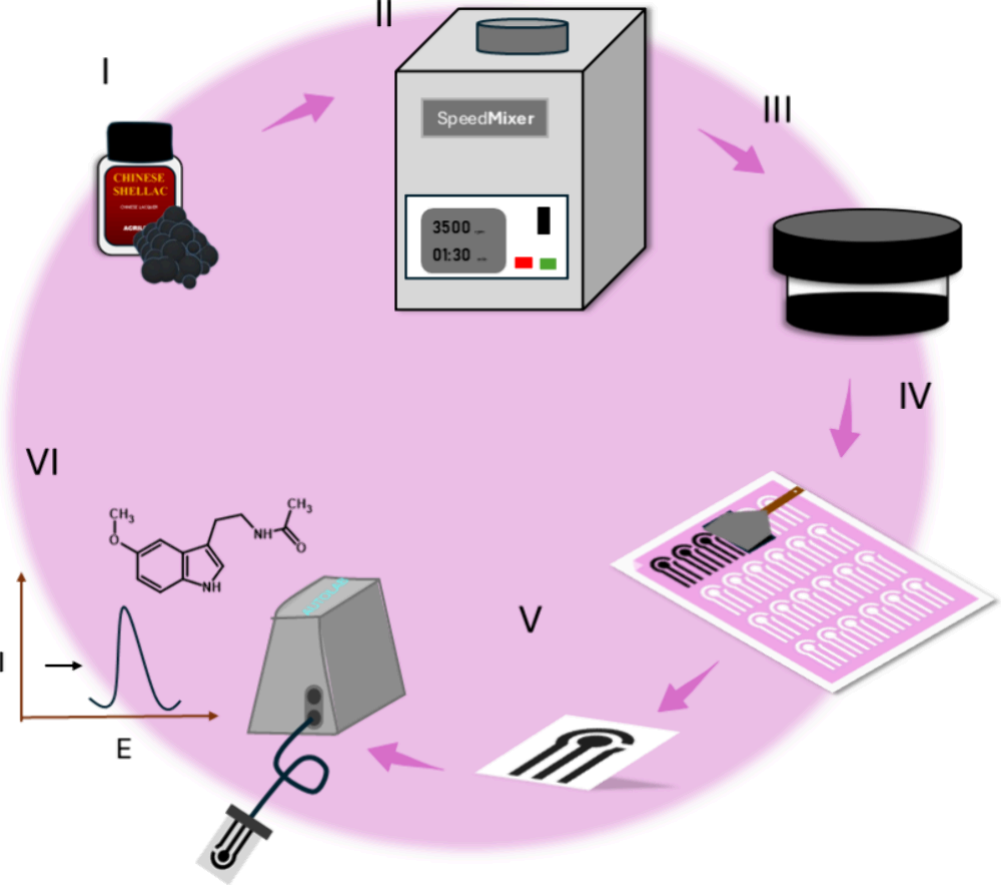
Preparation of the ChineseSHL-CB/acetate sensor. (I) Ink components; (II) ink homogenization; (III) conductive ink ready to use; (IV) printing of the electrodes using the screen-printing method; (V) screen-printed electrode; (VI) electrochemical detection of melatonin using the ChineseSHL-CB/acetate sensor.

### Morphological, Structural, Thermal, and Electrochemical Characterizations

The morphological characterizations were made using scanning electron microscopy (SEM) with a Prisma E microscope (Thermo Fisher Scientific) operating in high vacuum at 10 kV. For the structural characterizations were used a Fourier transform infrared spectroscopy (FTIR) with a Tensor II spectrophotometer (Bruker), at a resolution of 4.0 cm^–1^ and a scanning range from 400 to 4000 cm^–1^ (n = 64). KBr pellets in a 1:100 ratio was prepared for the conductive ink, CB and Chinese shellac. Crystallographic analyses were carried out using X-ray diffraction (XRD) with a Rigaku MiniFlex 600 X-ray powder diffractometer, employing a CuKα radiation source (λ = 0.15406 nm). Measurements were taken across an angular range of 2° to 90°, with a step size of 0.01° and a scanning speed of 10° min^–1^. Thermogravimetric analysis (TGA) was made in a nitrogen atmosphere with a flow rate of 20 mL min^–1^. The temperature was varied from 30 to 800 °C at a heating rate of 10 °C min^–1^. Electrochemical characterizations were performed by cyclic voltammetry (CV) and electrochemical impedance spectroscopy (EIS) with 1.0 mmol L^–1^ FcMeOH as the redox probe and 0.1 mol L^–1^ KCl as the supporting electrolyte. The EIS technique parameters applied were: frequency range of 1.0 × 10^5^ to 1.0 × 10^–^^1^ Hz, at 10 points per decade, 10 mV of amplitude, sinusoidal type waves and the potential applied were the half-wave potential (E1/2).

### Electrochemical Determination of Melatonin

The electrochemical determination of melatonin was investigated using SWV. Chemical and the specific parameters of the technique, such as pH, step (*s*), amplitude (*a*), and frequency (*f*), were optimized, in 100 μmol L^–1^ melatonin, in 0.2 mol L^–1^ PBS, pH 7.0. Once the analysis conditions were established, the calibration curve was constructed in the concentration range from 1.0 to 100 μmol L^–1^ to acquire analytical parameters for SWV, including the linear concentration range, limit of detection (LOD).

### Sample Preparation

The synthetic urine sample was prepared according to Campos Anderson and collaborators,^32^ with 0.25g of urea, 0.35g of KH_2_PO_4_, 0.73g of NaCl, 0.27g of CaCl_2_·2H_2_O, and 25g of NH_4_Cl in 250 mL of 0.2 mol L^–1^ PBS (pH 7.0). The melatonin sample was prepared using an addition and recovery method by using a melatonin stock solution.

## Results and Discussion

### Morphological Characterization

The morphological characterization of the conductive ink was performed by SEM for SPE, sanded acetate, and Chinese shellac film, in magnifications 500×, 1000× and 2000×, present in Figure 2. For the SPE surface, Figure 2C1–3, it is possible observed an irregular surface with a homogeneous dispersion of CB particles in the polymer matrix, as well as possible observed the formation of agglomerates, mainly in Figure 2C3. In all magnifications the presence of grooves caused by the sanding in the acetate substrate, Figure 2A. For the Chinese shellac sample, the magnifications showed a homogeneous surface, as shown in Figure 2B.

**Figure 2:**
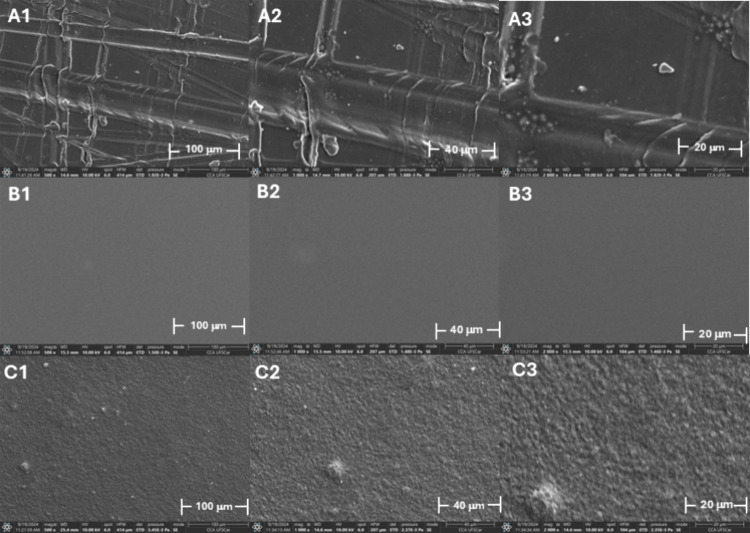
SEM images obtained for (A) sanded acetate, (B) Chinese shellac, and (C) screen-printed electrode (working electrode), with magnifications of (1) 500×, (2) 1000×, and (3) 2000×.

In Figure 3A, we can observe the spectra obtained by FTIR analysis for the CB (blue), Chinese shellac (red), and conductive ink (black) samples. In the Chinese shellac spectrum was possible to identify bands in 2800 cm^–1^ associated with symmetric and asymmetric stretching of C–H bonds.^33^ Also, can be observed an intense band in 1730 cm^–1^ of the anhydride symmetric stretching can be observed, which matches with the chemical structure of the polymer. Also, in the same spectrum, we observe bands in 1500–1450 cm^–1^ and 530 cm^–1^ related to the bonding of CH_2_ and CH_3_ bonds and the presence of long chains, respectively.^34^ The bands present in the range from 1600 to 400 cm^–1^, can be related to carboxylic, hydroxyl and ketone groups, which in the conductive ink can auxiliary on the dispersion of carbon material.^21^ For the CB, a band in 1600 cm^–1^, characteristic of C = C bonds,^35^ is observed. On the other hand, for the conductive ink, the spectrum obtained was similar to CB, where it is possible to observe some shellac bands from 1600 to 400 cm^–1^ with lower intensity. This result can be associated with some characteristics of the CB, such as the dark and low-density, which interfere in the analysis, besides the availability of CB on dispersion in ink. Furthermore, we observed in all analyses that the band in 3490 cm^–1^ was associated with the O–H bonds, which can be caused by humidity or O–H interactions.^36^

**Figure 3:**
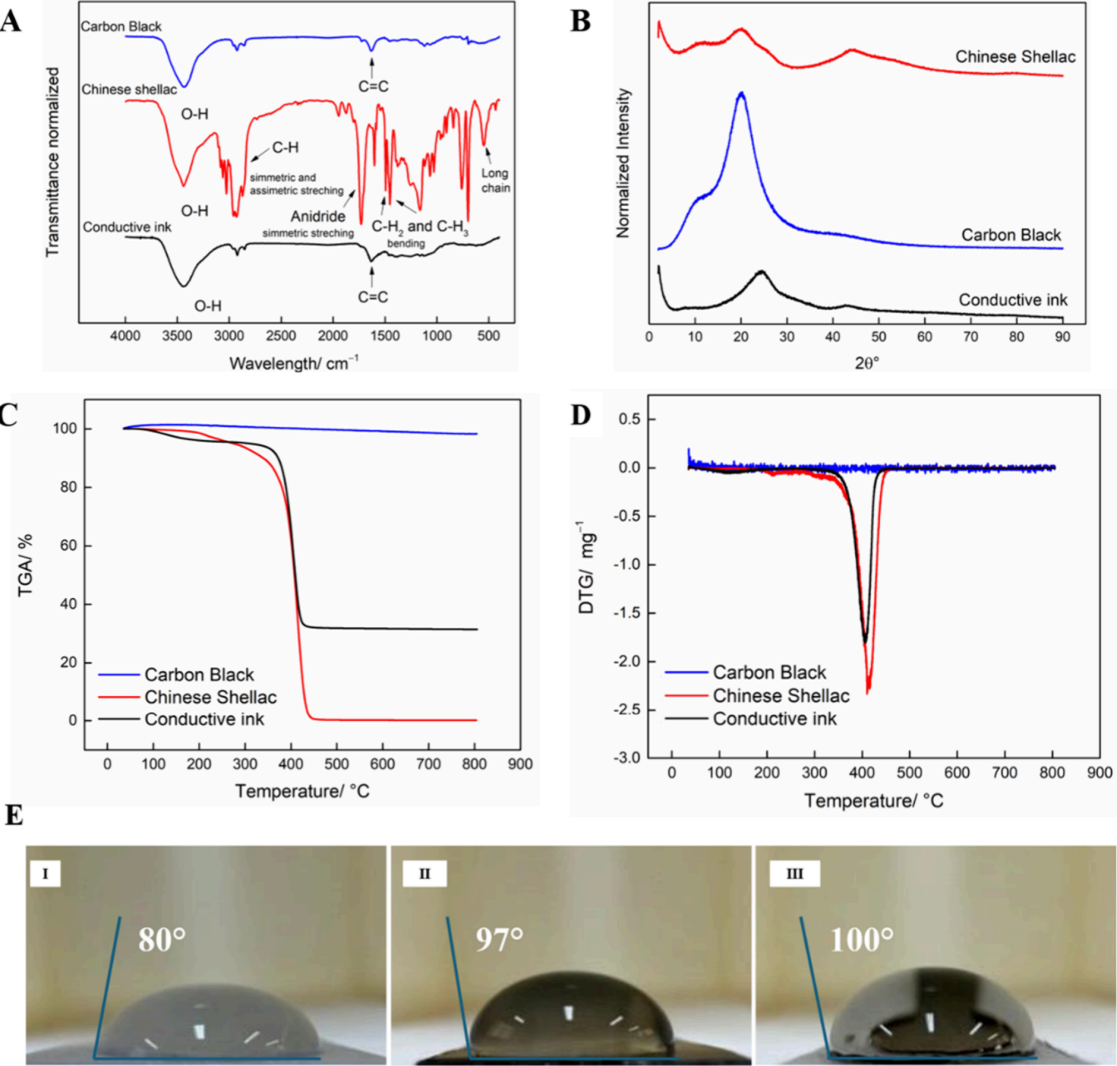
(A) FTIR spectra obtained from the samples of Chinese shellac (red), CB (blue), conductive ink (black), and amplified conductive ink spectrum inserted (black); (B) X-ray diffraction patterns of Chinese shellac (red), CB (blue), and conductive ink (black); (C) thermogravimetric analysis of CB (blue), Chinese shellac (red), and conductive ink (black) in percentage of total mass; (D) derivative thermogravimetry (DTG) of CB (blue), Chinese shellac (red), and conductive ink (black); (E) contact angle images of (I) sanded acetate; (II) conductive ink, and (III) ChineseSHL-CB/acetate sensor.

XRD patterns were obtained for the same samples analyzed in FTIR, being present in Figure 3B (red for Chinese shellac, blue for CB, and black for the conductive ink). The pattern of CB (blue line) presented an asymmetrical crystalline peak proximal to 20°, referent to the 002 plane, revealing the presence of characteristic planes of the amorphous material.^37^ Chinese shellac (red line), showed an amorphous pattern, with no characteristic peak of crystallinity, as seen in the literature for the maleic resin, Chinese shellac resin.^38^ Likewise, the conductive ink (black line) also presents an amorphous pattern, similar to the polymer pattern, as expected by its composition.

Furthermore, the TGA analysis were also made for the CB (blue), Chinese shellac (red) and conductive ink (black), as demonstrated in Figure 3C and D, for the percentage of degradation of these samples (Figure 3C) and its equivalent derivate (Figure 3D), to analyze the maximus degradation for each sample. In Figure 3C, it is possible to observe a small degradation in the conductive ink, starting at proximally 100 °C, possibly by the water evaporation. The conductive ink demonstrated a percentage of degradation of 69%, and the Chinese shellac of 99.68%, of their total mass, as seen in Figure 3C. Otherwise, the CB did not show a significative degradation in the thermal range analyzed. These results show that the degradation of the conductive ink is mostly referent to the presence of the Chinese shellac in its composition. In Figure 3D, it is more evident the maximus degradation peak in both samples, were the conductive ink exhibited the maximus degradation in 404 °C, and the Chinese shellac in 412 °C. Supposedly, this variation can be related to the heat concentrated for the presence of CB in the conductive ink, making its polymer degrade more easily.

Finally, the contact angle measurement was performed to evaluate the wettability of the ink on the 3-electrode system and substrate, providing information on the adhesion and spreadability of the ink formulation, which are essential factors for applications in flexible electronics and printed sensors. The measured contact angles were 80°, 97°, and 100° for the substrate, Figure 3E-I, conductive ink, 3E-II, and ChineseSHL-CB/Acetate sensor, 3E-III, respectively, indicating a varying degree of wettability. The substrate (Figure 3E-I) can be classified as a hydrophilic material, as its measured angle is lower than 90°. Otherwise, the conductive ink and device (Figure 3E-II and III), presented a similar angle, characteristic of hydrophobic materials, represented by their contact angle major to 90°. This similarity can be justified by the larger contact with the conductive ink than the substrate in the device system.^31^

### Electrochemical Characterization

To obtain the final ink formulation, different compositions were tested by varying the amount of carbon black (CB) relative to the shellac mass. The studied formulations contained 5%, 10%, 15%, and 20% (w/w) of CB. All formulations yielded conductive inks that were used to fabricate screen-printed electrodes. Cyclic voltammetry (CV) measurements were then performed using these electrodes in the presence of 0.1 mol L^–1^ FcMeOH in 0.1 mol L^–1^ KCl, at a scan rate of 50 mV s^–1^. As shown in the Supporting Information (Figure S2), increasing the CB content resulted in higher anodic and cathodic current responses, along with improved electrochemical behavior, evidenced by a decreased peak-to-peak separation. Based on these results, the selected formulation consisted of 83.33% Chinese shellac and 16.67% CB, corresponding to the 20% (w/w) composition.

To observe the electrochemical profile of the device, we performed CV measurements in the presence of 0.1 mol L^–1^ FcMeOH, in 0.1 mol L^–1^ KCl, at a scan rate (ν) of 50 mV s^–1^, showing the characteristic peaks of the electrochemical probe. In Figure 4, an oxidation peak can be observed at 160 mV and a reduction peak at – 52 mV. From this, a ΔEp of 238 mV was calculated, and a ratio of Ipa/Ipc of 1.22 was obtained, indicating a *quasi*-reversible behavior for the electrochemical process.^39^

**Figure 4:**
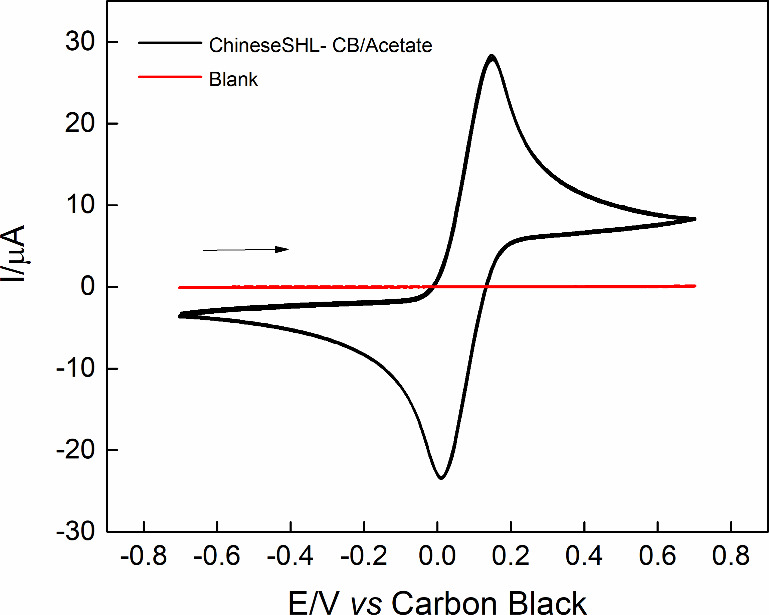
Cyclic voltammograms obtained by ChineseSHL-CB/acetate electrode in the absence (red) and presence (black) of 1.0 μmol L^–1^ FcMeOH, in 0.1 mol L^–1^ KCl; ν = 50 mV s^–1^.

Cyclic voltammetry was also performed at different ν, as present in Figure 5A, of 10, 25, 50, 75, 100, 125, 150, 175, and 200 mV s^–1^, using the electrochemical probe of 0.1 mmol L^–1^ FcMeOH, in 0.1 mol L^–1^ KCl. From the data presented in Figure 5A, it is possible to create and observe the linear relationship between the peak current and the increase in ν, Figure 5B. Thus, using the Randles-Ševčík equation for *quasi*-reversible systems:

$$\mathrm{Ip}=\pm (2,63\times 10^{5})\times A\times C\times D^{1/2}\times \nu^{1/2}\times n^{3/2}$$

Where I_p_ is the peak current (*A*), *n* is the number of electrons transferred, *A* is the electroactive area (cm^2^), *D* is the diffusion coefficient, *C* is the concentration of the electroactive species (mol cm^–3^), and ν is the scan rate (V s^–1^), it is possible to estimate the electroactive area of the electrode, which resulted in 0.180 cm^2^. Our result aligns with those reported by Orzari et al. (2024), who found an electroactive area of 0.172 cm^2^ in an ink made from CB and poly­(vinyl alcohol).^40^

**Figure 5:**
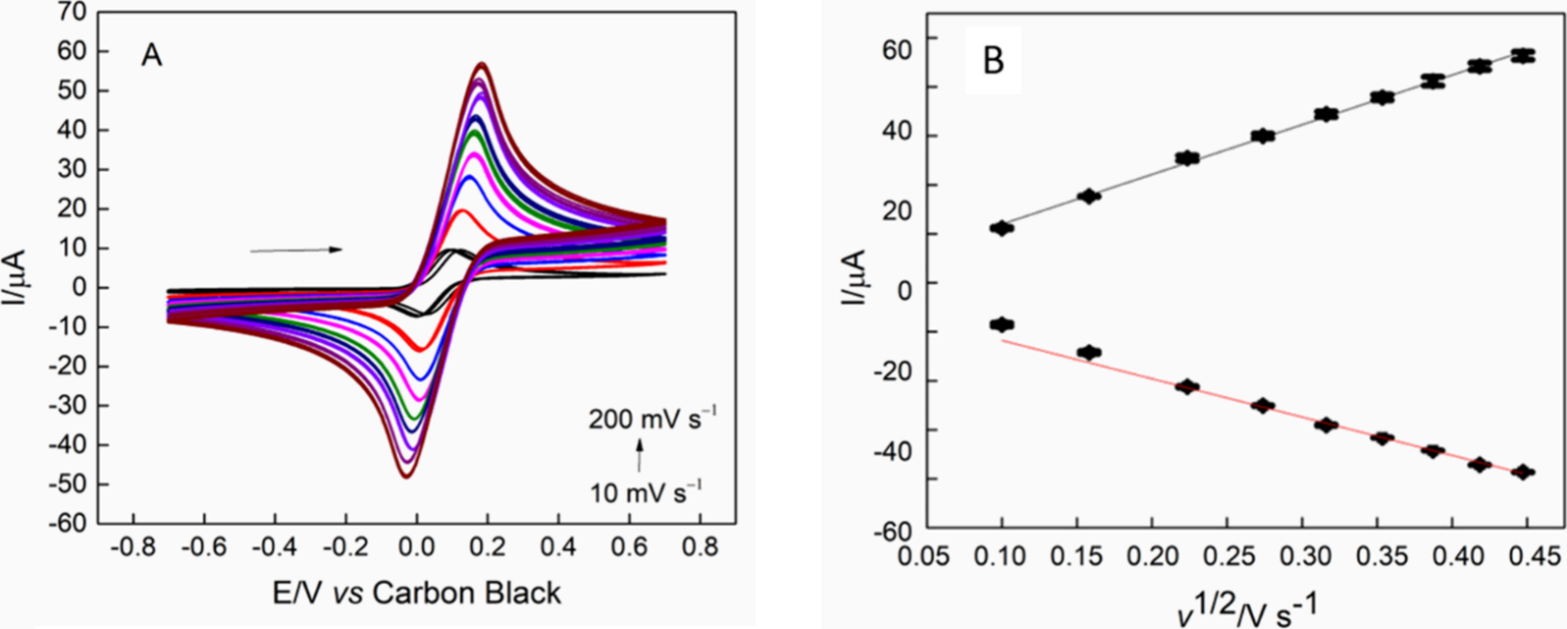
(A) Cyclic voltammograms obtained by ChineseSHL-CB/acetate electrode, in the presence of 1.0 mmol L^–1^ FcMeOH, in 0.1 mol L^–1^ KCl; ν = 10, 25, 50, 75, 100, 125, 150, 175, and 200 mV s^–1^. (B) Relation between *I* vs ν ^1/2^.

The electrochemical stability of the sensor was evaluated under different storage conditions by comparing the CV of freshly prepared electrodes and those stored for 14 days at room temperature or under refrigeration. As shown in Figure S3, all electrodes maintained a well-defined redox response, with only slight variations in peak current. These results indicate that the sensor preserves its electrochemical performance for at least 2 weeks under both storage conditions.

The EIS technique was used to analyze the electrochemical behavior of the sensor from the interaction of the surface and the solution, as shown in Figure 6. This data was obtained with the technique parameters shown in the experimental section, and the potential applied was the calculated *E*_1/2_ of 0.1 V. The Nyquist diagram showed a majority capacitive interaction in higher frequencies, related to the displacement parallel to the *x*-axis, followed by two decrescent capacitive interactions until the mass transport is majority controlled by diffusion, represented by the Warburg impedance, present in the circuit. So, this device behavior is represented by a modification of the Randles circuit, where two pairs of resistance and capacitance are present, attributed to two different phases. The first phase can be attributed to a thin layer of the shellac, and the second to the CB and Chinese shellac interaction.

**Figure 6:**
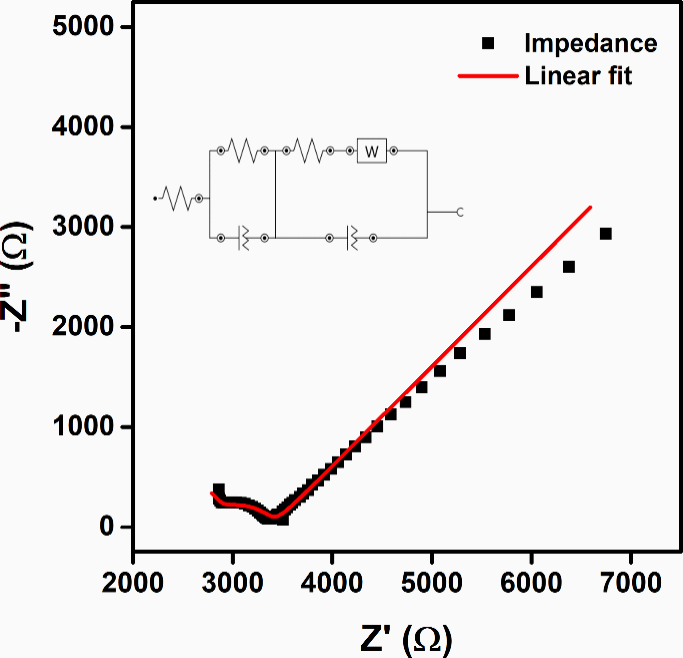
Nyquist diagram obtained by ChineseSHL-CB/acetate electrode (black), in the presence of 1.0 mmol L^–1^ FcMeOH, in 0.1 mol L^–1^ KCl; E1/2 = 0.1 V, equivalent circuit inserted: [R­(RQ)­([RW]­7Q)].

### Electrochemical Behavior of Melatonin

The ability of melatonin to cross biological barriers, such as the blood-brain barrier, gives it neuroprotective and antioxidant properties. Because of this, it becomes a promising therapeutic target for various neurological conditions, ranging from sleep disorders to neurodegenerative diseases.^41^

The electrochemical behavior of melatonin was initially evaluated by cyclic voltammetry in the absence and presence of 100 μmol L^–1^ melatonin, in 0.2 mol L^–1^ PBS (pH = 7.0). In Figure 7A, an oxidation peak is observed at approximately 0.8 V. The response mechanism of melatonin is illustrated in Figure 7B, where the substance undergoes an irreversible oxidation reaction, likely involving the transfer of two electrons and one proton, resulting in the formation of a radical cation. This pattern of behavior has been previously described in the literature by Camargo et al.^42^ and Freitas et al.^43^

**Figure 7:**
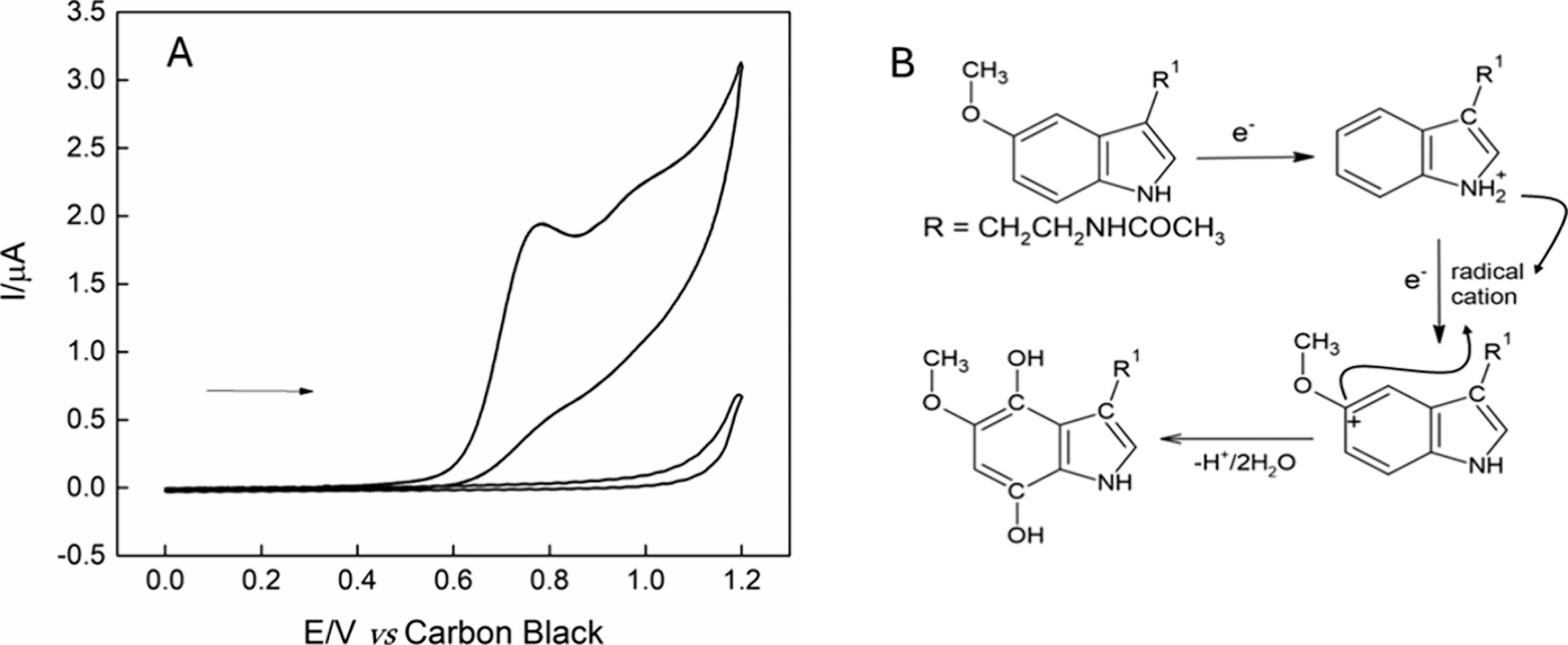
(A) Cyclic voltammograms obtained by ChineseSHL-CB/acetate electrode in the absence and presence of 100 μmol L^–1^ melatonin, in 0.2 mol L^–1^ PBS solution (pH 7.4); ν = 100 mV s^–1^. (B) Proposed oxidation mechanism of melatonin.

The melatonin determination was carried out using the SWV, to define the best conditions for melatonin quantification. Optimization studies of chemical and specific parameters of the technique were conducted, with the results presented in the Supporting Information, as Table S1. Using the SWV technique, the behavior of the analyte was studied at different pH values in PBS, in the pH range of 5.0 to 8.0. This study indicates that the peak potential tends to increase as the pH increases. Therefore, to achieve greater sensitivity of the sensor, the ideal pH of the supporting electrolyte was determined to be 7.0, which was chosen for future studies. The specific parameters of the technique that influenced the current response in SWV were also evaluated to obtain the highest currents and best voltammetric profiles. The parameters analyzed were *a*, *f*, and *s*. Its studied range for *a* was from 10 mV to 100 mV, *f* from 10 to 100 Hz, and *s* from 1 mV to 10 mV. The optimized values found for these parameters were: *a* = 80 mV, *f* = 20 Hz, and *s* = 6 mV, present in Table S1.

### Voltammetric Determination of Melatonin

With the defined parameters, an analytical curve was constructed using SWV by varying the melatonin concentration, using 1.0, 3.0, 5.0, 7.0, 10.0, 30.0, 50.0, 70.0, and 100 μmol L^–1^, in a 0.2 mol L^–1^ PBS (pH = 7.0). In Figure 8A, the square wave voltammograms showed a progressive increase in the anodic current signal with the increase in concentration, demonstrating a linear relationship between the peak current and the melatonin concentration within the studied range, Figure 8B. From this relationship, the linear equation obtained was I = 0.01883 C_(Melatonin)_ – 2.92533 × 10^–8^, with an r^2^ = 0.996. The LOD was calculated by 3 × RSD of blank divided by slope, resulting in 0.11 μmol L^–1^. A slight displacement for higher potentials can be observed, with the increase of concentration, which is possibly associated with an adsorption process. Additionally, studies on repeatability and reproducibility were performed, with results showing an RSD = 3.81% (n = 5) and RSD = 7.80% (n = 5), respectively.

**Figure 8:**
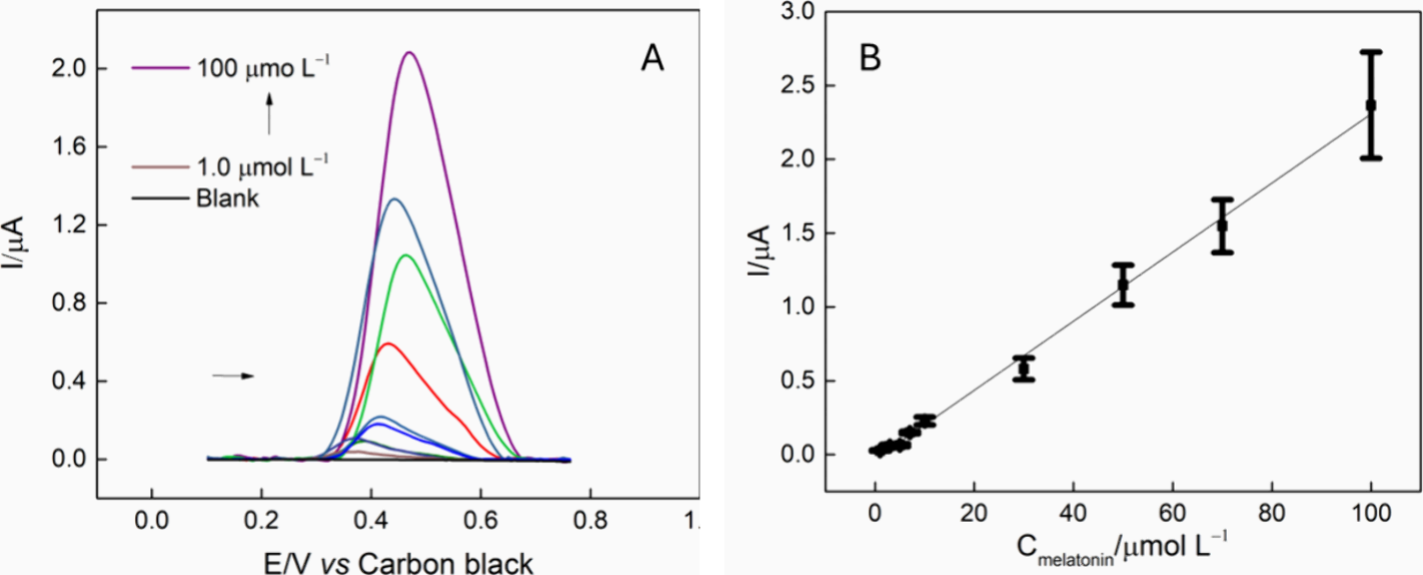
(A) Square wave voltammograms obtained by the ChineseSHL-CB/Acetate electrode in the absence, and presence of melatonin, varying the concentration from 1.0, 3.0, 5.0, 7.0, 10.0, 30.0, 50.0, 70.0, and 100 μmol L^–1^, in 0.2 mol L^–1^ PBS (pH = 7.0); parameters: *a* = 80 mV, *f* = 20 Hz, and *s* = 6.0 mV. (B) Relation current vs concentration of melatonin.

The determination of melatonin was performed in synthetic urine samples using the addition and recovery method. Table 1 presents the results obtained, with values ranging from 86.7% to 110%. This demonstrates the feasibility of using the proposed device for the detection of the hormone in this biological fluid.

**Table 1:** Determination of Melatonin in Synthetic Urine

sample	added (μmol L^–1^)	recovered (μmol L^–1^)	recovery ± (%)
**1**	5.0	5.36 ± 0.025	107 ± 4.8
**2**	10	11.1 ± 0.04	110 ± 5.5
**3**	10	9.61 ± 0.039	96.1 ± 5.2
**4**	100	110.1 ± 0.02	110 ± 11

To evaluate the selectivity of the proposed sensor, interference studies were carried out using common biomolecules found in biological fluids, namely uric acid, urea, dopamine, and ascorbic acid. Although some variations in signal intensity were observed, the sensor maintained a distinguishable response in the presence of all interferents. The highest interference was recorded for ascorbic acid (33.64%) and uric acid (28.24%), while urea and dopamine exhibited the lowest interference of 9.61% and 9.01% respectively. Detailed results, including average current responses, standard deviations, and relative deviations, are presented in Table S2. Also, to decrease this interference, electrode modification should be performed, which can be used in further works.

For a fair comparison, we present some works from the literature, also for electrochemical melatonin determination, presented in Table 2. For instance, the device produced by Freitas et al.,^43^ a disposable self-adhesive inked paper electrode, exhibited a higher LOD, if compared with this work, and its linear range starts also at higher values, if compared with all the analyzed devices. Otherwise, the Camargo et al.^42^ showed the lower LOD, followed by the one developed in this work, with a waterproof paper-based electrode. And the Gomez et al.,^44^ covered a broader linear range, using CNTs and graphene-based, though with a higher LOD. Satianram et al.^13^ developed a Tween-coated h-BDD modified screen-printed electrodes for melatonin, and showed a lower LOD in relation the other works cited in the table. Some sensors used modified glassy carbon electrodes for the determination of melatonin, as Dung et al.,^45^ who present a wide linear range for detection, and Lete et al.^29^ who obtained a linear range with lower concentration values of melatonin. However, both of them are modified with metallic nanoparticles, as FeCo alloy nanoparticles and Au nanoparticles, respectively. So, it is possible to conclude that the performance of ChineseSHL-CB/Acetate, developed in this work, can be considered analytically appreciable when compared to other systems. Therefore, we present a new sensor, which has great characteristics such as a simple fabrication process and potential for widespread use.

**Table 2:** Comparison of Proposed Electrodes for Melatonin Determination

sensors	linear range (μmol^–1^)	LOD (μmol^–1^)	reference
Gr-Av[Table-fn t2fn7]	10–100	0.47	^43^
GPT/WPE[Table-fn t2fn8]	0.8–100	0.033	^42^
CNTs and graphene-based CSPE[Table-fn t2fn9]	5.0–300	1.1	^30^
Tween/h-BDD/SPE[Table-fn t2fn10]	0.057–10.00	0.017	^13^
	10.00–200.00		
CNFs/FeCo[Table-fn t2fn11]	0.08–400.00	0.0027	^45^
SNGCE/AuNP[Table-fn t2fn12]	0.02–0.3	0.0084	^29^
	0.5–20.00		
ChineseSHL-CB/acetate	1.0–100	0.107	this work

a Disposable self-adhesive inked paper electrode.

b Waterproof paper electrodes.

c Modified screen-printed electrode with carbon nanotubes and graphene.

d Screen-printed electrode with highly boron-doped diamond coated with Tween.

e Electrode based on carbon nanofibers embedded with FeCo alloy nanoparticles.

f Sensor based on a Sonogel-carbon electrode enriched with gold nanoparticles.

## Conclusions

A new screen-printed electrochemical sensor has been successfully developed, offering a rapid, sensitive, and practical approach for detecting melatonin. The fabrication process of the sensor was notably straightforward and cost-efficient, leveraging a conductive ink formulated from Chinese shellac and carbon black (CB). This ink was engineered to possess optimal viscosity for printing, thereby simplifying the production process and reducing overall manufacturing costs. The sensor demonstrated a broad and effective detection range, reliably measuring melatonin concentrations from 1.0 to 100 μmol L^–1^. Employing square wave voltammetry (SWV), the sensor exhibited excellent performance in analyzing biological samples, specifically synthetic urine. Accuracy was assessed using the standard addition and recovery method, yielding recovery rates between 86.7% and 110%, which confirms the method’s robustness and reliability. In addition to its sensitivity and affordability, the device showed strong stability, repeatability, and reproducibility across multiple tests. These characteristics highlight its significant potential for real-world applications in clinical diagnostics and biological fluid analysis, making it a promising tool for consistent and dependable melatonin detection.

## Supplementary Material



## Notes

Photo of the proposed electrodes, cyclic voltammograms from the stability study, cyclic voltammograms showing the percentage contents of the ink, tables of studied parameters, and a table of the interference study (PDF)
