# Supplementary material for: Disposable Printed Electrode Made with Chinese Shellac and Carbon Black for Melatonin Detection
Source: ACS Meas Sci Au. 2025 Jul 18;5(4):572–80. doi: 10.1021/acsmeasuresciau.5c00056 (PMC12371578; doi:10.1021/acsmeasuresciau.5c00056)
Supplement: Supplementary file 1 [file tg5c00056_si_001.pdf]

Disposable printed electrode made with Chinese shellac and Carbon black for melatonin detection

Ana Luiza Molina De Cezar[a]; Rafaela Cristina Freitas[a]; Amanda Neumann[a]; Bruno Campos Janegitz[a*]

[a]*Laboratory of Sensors, Nanomedicine, and Nanostructured Materials, Universidade Federal de São Carlos, 13604-900, Araras São Paulo, Brasil*

Corresponding author: brunocj@ufscar.br

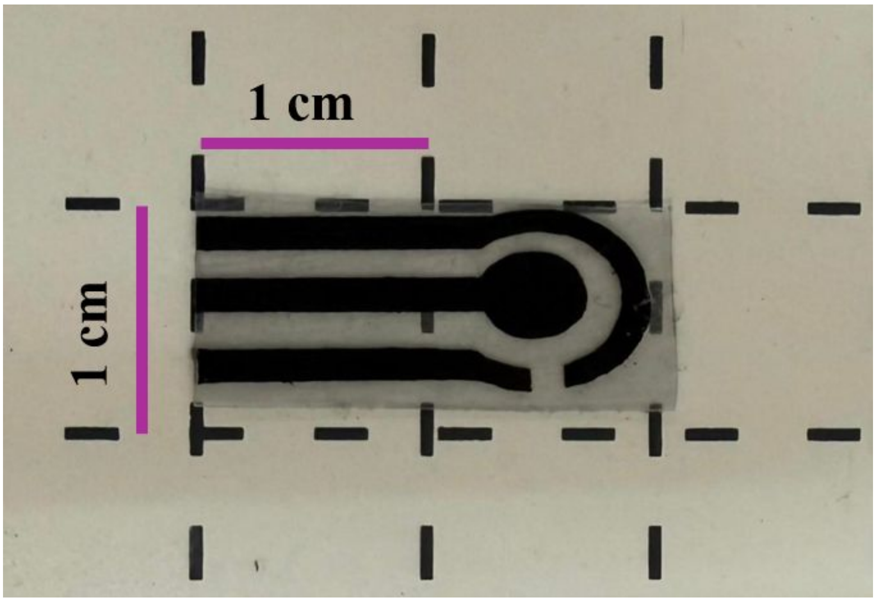

Figure S1. Image of ChineseSHL-CB/Acetate sensor ready to use

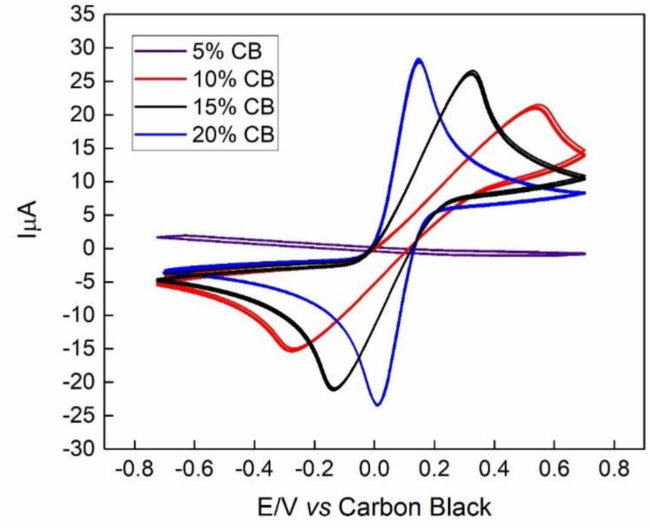

Figure S2. Cyclic voltammograms obtained using ChineseSHL-CB/Acetate electrodes prepared with different carbon black concentrations: 5% (purple line), 10% (red line), 15% (black line) and 20% (blue line), in the presence of 1.0 μmol L^{-1} FcMeOH and 0.1 mol L^{-1} KCl; scan rate: 50 mV s^{-1}.

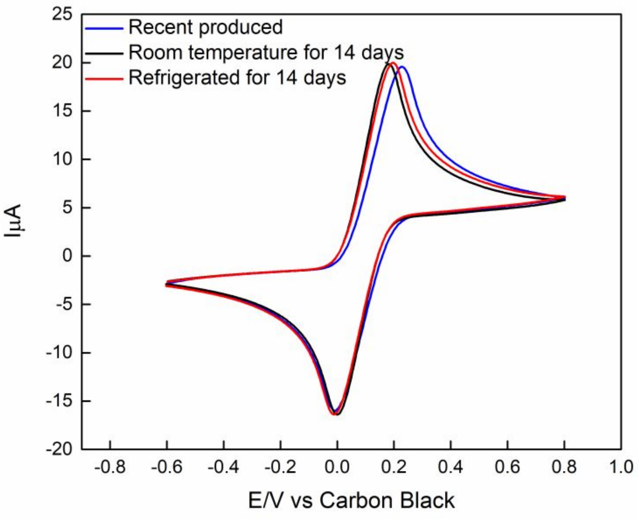

Figure S3. Cyclic voltammograms of the electrode recently produced (blue), stored at room temperature for 14 days (black), and refrigerated for 14 days (red). Measurements were carried out in the presence of 1.0 μmol L^{-1} FcMeOH in 0.1 mol L^{-1} phosphate buffer (pH 7.0) at a scan rate of 50 mV s^{-1}. The results demonstrate the electrochemical stability of the sensor under different storage conditions.

Table S1. Optimized values of pH and the technique parameters

Parameters	Studied values	Optimized values
**pH**	5.0 - 8.0	7.0
**Amplitude (mV)**	10 - 100	80
**Frequency (Hz)**	10 - 100	20
**Step (mV)**	1.0 - 10	6.0

Table S2. Interference of common biomolecules on the electrochemical response of the proposed sensor.

Interferent	Mean Current (A)	Standard Deviation (A)	Interference (%)
Uric Acid	1.47×10^{-6}	1.86×10^{-7}	28.24
Dopamine	1.04×10^{-6}	1.79×10^{-7}	9.61
Ascorbic Acid	9.01×10^{-7}	3.11×10^{-8}	33.64
Urea	1.53×10^{-6}	3.58×10^{-8}	9.01
